# Medico-Legal Facts and Testimony in the Trial of Henry C. Hendryx, for the Murder of His Wife

**Published:** 1877-09

**Authors:** J. C. Young

**Affiliations:** Cuba, Allegany Co.


ART. III.—*Medico-Legal Facts and Testimony in the Trial of Henry C. Hendryx for the murder of his wife.* By J. C. YOUNG, M. D., of Cuba, Allegany Co.

In placing before the readers of the BUFFALO MEDICAL AND SURGICAL JOURNAL, the medico-legal testimony before the coroner's jury, and subsequently on the trial of Henry C. Hendryx for the murder of his wife, it may be of some interest and guide to the medical profession to learn a brief history of the surrounding circumstances of the case, although with the motive that a person may have for committing crime, the medical profession as such are not concerned. Mr. Hendryx was a farmer living in the town of Cuba, Allegany Co., N. Y. Had been a soldier, and like all soldiers was accustomed to the use of fire arms. He owned a revolver, and the ball found in the abdomen of Mrs. Hendryx after death, was fired from a weapon that would carry a ball the size of the one owned by him. He had been married about eleven years, their family consisted of an only child, a boy nine years of age. About two o'clock A. M., the morning of July 7th, 1876, Mrs. Hendryx was shot in her bed when asleep, and died from the effects of said injury, July 13th, 1876. Mr. Hendryx was wounded in the left thigh, and thumb and fore finger of the left hand. The previous character of Mr. Hendryx had always been above reproach, and his explanation of the affair, received all the credit that any explanation could, as far as it was consistent with the absolute facts in the case. It was substantially as follows: He was awakened in the night by a noise in his bedroom, or near there, and as he awoke he thought he saw some one near the bed, and as he went to get up a shot was fired that proved to be the fatal shot to his wife. He went immediately out into the front room to the door which was closed, but unlocked by the burglars, opened it, and another shot was fired which wounded him, and the burglars made their escape. Said he could not remember that more than two shots were fired, although there might have been the third one. When day-light came he found his pocket-book in the front yard, and the money, from eight to ten dollars gone. Himself and wife had always lived happily together, and it seemed impossible for him to do such a deed and suspicion fastened on him only, when it seemed impossible for the work to have been done as it was by burglars.

1st. His stories were very conflicting, although it must not be expected that any one, under the circumstances in which Mr. Hendryx was placed could tell it very many times every day, and always get it just alike, or that so many different individuals would understand it alike.

2d. He had solicited and obtained an insurance on the life of his wife some four weeks previous for $2000, the same on himself against accidents. His interest in the insurance lay in the death of his wife to obtain that and his own to receive an injury and compensation fee for loss of time. But this of itself would have excited no suspicion, as his wife had no hesitancy in his procuring an insurance on his life.

3d. About the first of June, 1876, a cousin of Mr. Hendryx came to make it her home at his house, and as she had lived near them for a few months previous, had been quite intimate with both Mr. and Mrs. Hendryx. After she made it her home there Mr. Hendryx and cousin were quite frequently seen riding together, and unacompanied by Mrs. Hendryx, but previous to the tragedy their familiarity excited no remarks from the public in general.

4th. They were in each others society during the brief illness of Mrs. Hendryx, enough so that suspicion was arroused by the attendants of Mrs. Hendryx. But this did not prove that some one else other than the husband could not be the perpetrator of this crime, for he was with his wife considerable during her illness, and at times manifested grief.

5th. He fired all the shots out of his revolver in the morning before any one arrived at the house. Said to have done so to alarm the neighbors. Also stated that previous to the arrival of any one, his wife requested him to marry his cousin, as she would make a good mother to the little boy. It should be remembered that no one was staying at the house that night, only the family, and the testimony of the little boy was not brought out on the trial, and before the coroner's jury nothing of importance was elicited that would throw any light upon the tragedy.

The most positive evidence in the case pointing to Mr. Hendryx as the quilty man was the wounds on himself and a hole in his shirt; also the position that the assailent must have been in to have shot Mrs. Hendryx as she was shot. The medical testimony was by far the most *convicting*, and also the most conflicting in character.*

* The detail of medical evidence is too long for publication. The question of entrance and exit of ball, together with possible deflection was the subject of a vast amount of widely differing medical testimony all going to show how doctors are liable to disagree. The jury however, agree that the husband was guilty of murder in the second degree, and prisoner was sent to Auburn for life. The verdict was an absolute absurdity. The prisoner was either innocent, or guilty of premeditated, wilful, atrocious murder. There could have been no "second degree" in the case. Conflicting medical testimony, and absurd and wholly irrational verdict is a fair and condensed description of the case, which is too extensive for our pages.

